# Sequencing AI Automation and Data Interoperability in Oncology Using a Scenario-Planning Framework Coupled With Discrete-Event Simulation: Proof-of-Concept Study

**DOI:** 10.2196/92642

**Published:** 2026-05-25

**Authors:** Peter May, Sabine D Brookman-May, Edward Garrahy, Johannes von Büren

**Affiliations:** 1 Department of Medicine III, School of Medicine and Health, Technical University of Munich, Ismaninger Str. 22, Munich, Bavaria, 81675, Germany, 49 89-4400 ext 8753; 2 Department of Urology, Ludwig-Maximilians-Universität München, Munich, Bavaria, Germany; 3 Wellster Healthtech Group, Munich, Germany; 4 Sidekick Health Germany GmbH, Hamburg, Germany

**Keywords:** medical futures studies, artificial intelligence, AI, data interoperability, scenario planning, discrete-event simulation

## Abstract

**Background:**

As oncology workflows integrate increasingly autonomous artificial intelligence (AI) agents, health systems face uncertainty regarding operational impacts. Traditional linear forecasting methods fail to capture second-order effects such as governance saturation, induced demand, and bottleneck migration. To navigate this complexity, the emerging field of medical futures studies requires methodologies that bridge qualitative strategic foresight with quantitative operational modeling. These system-level dynamics directly influence timely diagnosis, treatment delays, and overall health system resilience.

**Objective:**

This study aimed to develop a proof-of-concept framework coupling qualitative scenario planning with computational discrete-event simulation to stress-test oncology AI adoption strategies.

**Methods:**

We defined a strategic state space using 2 orthogonal axes, AI automation intensity and data interoperability, resulting in 4 distinct futures scenarios. We translated these qualitative narratives into a quantitative discrete-event simulation model of a 3-year operational horizon. The model quantified system performance (referral-to-treatment interval [RTTI] and throughput), volatility, and resource constraints across different adoption trajectories.

**Results:**

The scenario-planning phase yielded 4 operational archetypes (analog oncology, automation islands, interconnected clinicians, and AI-orchestrated care) with distinct constraints, risks, and failure modes. In the simulation, the fully integrated scenario maximized capacity (1244, SD 21.4 patients per year) and halved the mean RTTI to 14.9 (SD 0.3) days, a magnitude comparable to major pathway redesign interventions. Isolated automation without data infrastructure led to reduced system performance, increasing RTTI by 26% (37.1, SD 1.3 days) and reducing throughput to 647 (SD 10.1) patients per year due to administrative governance saturation. The model illustrated a structural bottleneck migration: successful upstream AI adoption shifted binding constraints from diagnostic scanners to downstream chemotherapy infusion units, whereas missing data interoperability resulted in governance constraints. Pathway optimization analysis indicated that a coordinated strategy prioritizing early improvements in data interoperability reduced transition volatility compared to an automation-first approach.

**Conclusions:**

Integrating qualitative scenario planning with quantitative simulations enabled a systematic evaluation of oncology AI adoption strategies. As a proof of concept, it offers a replicable framework for health leaders to model future scenarios of digital transformation in times of high uncertainty. Subsequent work should expand this methodology to incorporate financial and health equity dimensions, establishing simulation-based scenario planning as an important tool in medical futures studies.

## Introduction

Modern oncology faces an increasing gap between therapeutic potential and operational capacity. While precision medicine expands, care delivery remains constrained by diagnostic throughput, pathology backlogs, and the cognitive load of multidisciplinary coordination. At the same time, clinical artificial intelligence (AI) is evolving from narrow, task-specific applications toward workflow-native systems [1]. This next generation includes large language models capable of complex documentation and eligibility screening alongside emerging agentic AI systems designed to execute multistep planning with minimal human intervention [2-6]. Recent reviews describe the rapid proliferation of these tools in oncology yet consistently highlight that their translation into practice is constrained not by algorithmic performance but by integration challenges, data quality, and safety governance [7-13].

The impact of AI in complex adaptive systems such as oncology is rarely linear; local efficiency gains often destabilize rather than improve system-level performance. There is evidence suggesting that altering information flows, which is heavily dependent on robust data interoperability, can shift use patterns and clinical outcomes [14-16]. However, these integration efforts often trigger higher-order effects such as bottleneck migration and induced demand, which are usually not captured by model-centric evaluations. Furthermore, as sociotechnical complexity grows, regulatory frameworks are becoming more relevant [17,18]. New standards such as DECIDE-AI (Developmental and Exploratory Clinical Investigations of Decision Support Systems Driven by Artificial Intelligence), the European Union AI Act, and the Food and Drug Administration Good Machine Learning Practice now specify context-aware assessment and life cycle oversight [19]. Consequently, governance moves from a background administrative activity to a potential capacity-limiting resource, influencing whether automation yields genuine access gains or merely introduces new operational friction.

Addressing these nonlinear dynamics requires methodologies capable of navigating uncertainty, a domain where health care has historically underused strategic foresight [20-22]. Scenario planning, a mature management science technique, offers a structured approach to reasoning through such strategic uncertainty [23]. While futures studies in adjacent fields such as biobanking and education demonstrate how governance and incentives determine whether technical visions become reality, these methods are rarely applied to operational capacity planning [24,25].

To bridge this gap using medical futures thinking, we coupled qualitative scenario planning with computational discrete-event simulation (DES) in this study [26]. By translating plausible adoption trajectories into quantitative models, we analyzed how variables such as AI automation intensity and data interoperability interact to produce higher-order effects, including governance saturation, false positive rates, and volatility. This paper presents a proof-of-concept framework for governments and health care executives to plan oncology AI adoption, shifting the focus from static operational forecasts to the dynamic management of structural transformation under uncertainty. In the following sections, we first describe our scenario design and simulation model; then present comparative results across scenarios; and, finally, discuss strategic implications.

## Methods

### Study Design and Framework

We integrated qualitative scenario planning with DES to stress-test oncology operations against alternative adoption futures. Scenario planning defines plausible strategic futures, whereas DES evaluates how those futures propagate through operational constraints over time. To structure the uncertainty space, we first identified the 2 most decisive yet independent drivers of digital transformation: AI automation intensity (*A*), representing the degree of algorithmic task substitution in clinical and administrative workflows, and data interoperability (*I*), representing the friction of data transfer, provenance, and reuse across system boundaries. By treating these variables as orthogonal rather than correlated, we defined 4 operational quadrants in the 2 × 2 scenario analysis and qualitatively explored their outcomes and risk profiles.

### DES Model

To quantify operational implications, we constructed a DES model tracking individual patient trajectories through a resource-constrained oncology system over time. Implemented in SimPy (Python version 3.14; Python Software Foundation), the model represented a generic high-volume cancer center as a network of finite queues (eg, scanners, pathologists, oncologists, and treatment chairs). We selected resource capacities, service times, and failure rates based on standard oncology operation principles to create a representative, capacity-constrained baseline (the full script can be found in Multimedia Appendix 1). This stylized parameterization was chosen to isolate the mechanistic effects of variables *A* and *I* on system flow rather than fit the historical data of a single institution. Consequently, the results illustrate directional trends and structural vulnerabilities rather than precise, generalizable operational forecasts.

The simulation ran for 3 years (1095 days) to ensure statistical convergence of queue dynamics and capture the full variance of patient pathways; a 150-day warm-up period was used to reduce initialization bias. Patient agents were generated stochastically based on the 3 most common tumor types (lung, breast, and colorectal) and disease stages (early vs late), flowing through a pathway that encompasses administrative processing, diagnostic imaging, biopsy, pathology (with probabilistic rework loops), molecular testing, multidisciplinary team coordination, and multimodal treatment initiation (surgery, chemotherapy, and radiotherapy). A calendar engine distinguished between standard clinical shifts and continuous laboratory operations, enforcing atomic task logic to prevent procedural fragmentation across shifts.

The model was governed by the 2 exogenous variables (*A* and *I*) normalized from 0.0 to 1.0. Workflow durations (*T*) for clinical tasks were calculated dynamically as follows: *T*_task_ = *T*_base_ × (1 – α · *A*) × (1 – β · *I*), where α and β represent the susceptibility of the task to automation and data integration, respectively.

### Operational Mechanisms

The simulation incorporated 8 coupled mechanisms to capture higher-order behaviors beyond linear efficiency gains (Table S1 in Multimedia Appendix 2). These mechanisms captured 3 core operational dynamics: administrative friction, where high AI automation intensity without sufficient data interoperability triggers manual verification and shifts bottlenecks to administrative staff; induced demand, where highly sensitive AI triage increases false positive rates and generates additional downstream biopsies; and pathway shifts, where faster upstream diagnosis accelerates patient arrivals at downstream treatment centers and alters multidisciplinary coordination requirements. Each mechanism was operationalized through parameterized interactions governing queue progression, resource availability, and downstream demand propagation.

### Experimental Design and Analysis

We executed a comprehensive parameter sweep across the *A* × *I* state space with multiple stochastic replications per coordinate to generate uncertainty surfaces. Primary outcome measures included the mean referral-to-treatment interval (RTTI; defined as the simulated time in days from a patient’s system entry at intake to the start of their first definitive treatment event), system throughput (patients per year), governance event density, and resource use profiles.

### Transition Pathway Optimization

To identify the most robust adoption sequence from *A*=0 and *I*=0 to *A*=1 and *I*=1, we modeled the simulation grid as a weighted directed graph. Transitions were restricted to orthogonal movements (increasing either *A* or *I* but not both simultaneously) to simulate realistic, incremental implementation capacity. A composite cost function was applied to determine the trajectory that minimizes the cumulative operational friction. The cost function was defined as the sum of 4 equally weighted (*w*=0.25) normalized components: mean RTTI, RTTI SD, inverse throughput volume, and throughput SD. We applied the shortest path algorithm by Dijkstra [27] to minimize this cumulative operational cost, implemented using the NetworkX library [28]. Conceptually, this treated the adoption journey as a risk landscape navigation problem, allowing us to mathematically identify the specific sequence of investments that reaches full integration while maximizing efficiency, throughput, and stability. This optimal path was benchmarked against 2 corner strategies: automation first (fully maximizing *A* before *I*) and data interoperability first (fully maximizing *I* before *A*).

### Ethical Considerations

Ethical review was not applied for. Because this research relied entirely on a theoretical DES using synthetic parameters and did not involve human participants or identifiable personal health information, it was exempt from institutional review board oversight in accordance with the statutes of the Ethics Committee of the Technical University of Munich [29].

## Results

### Operational Landscape of AI Adoption

**Figure 1.**
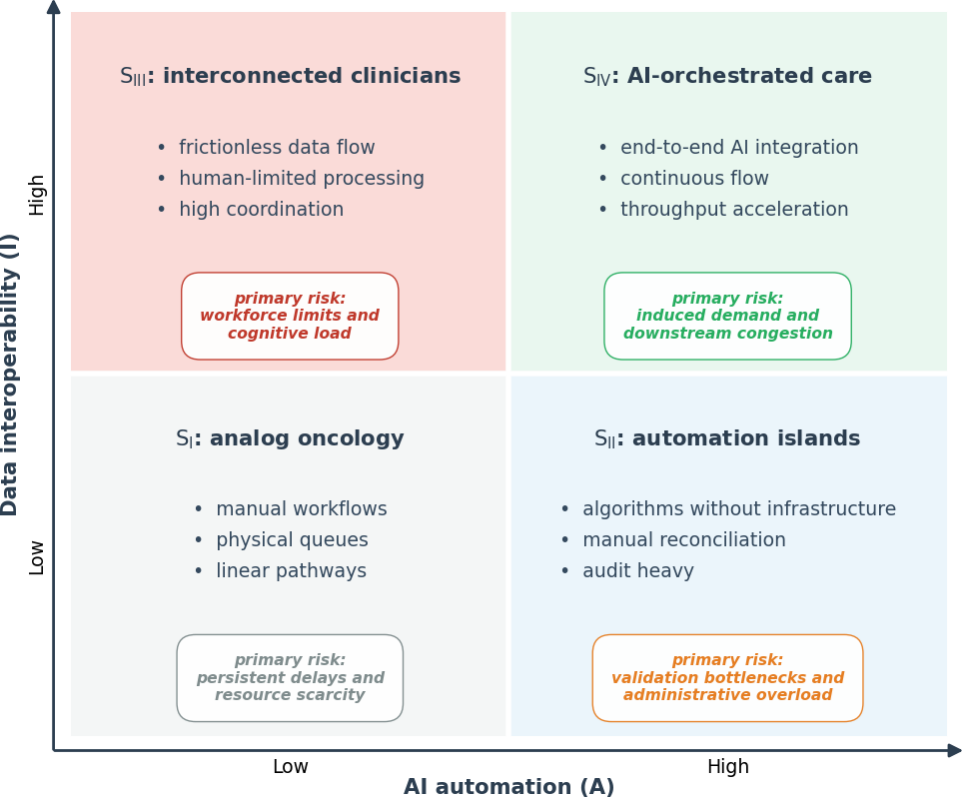
Conceptual 2 × 2 scenario analysis matrix for artificial intelligence (AI) adoption in oncology. The strategic state space is defined by AI automation intensity (x-axis) and data interoperability (y-axis). The intersection yields 4 distinct operational scenarios (*S*_I-IV_). Each quadrant details the anticipated dominant workflow characteristics, structural constraints, and primary expected operational risk.

The scenario-planning phase defined a 2D parameter space using AI automation (A) and data interoperability (I). This yielded 4 scenarios (S_I-IV_) with corresponding operational constraints and failure modes (Figure 1).

The analog oncology scenario (S_I_: low *A* and low *I*) represents the baseline state, where capacity is dictated by physical constraints and linear, manual workflows. Here, digitization is localized, and delays are governed by visible queues in administration and diagnostics.

Increasing only AI automation (S_II_: high *A* and low *I*) creates automation islands. Here, algorithmic deployment outpaces data interoperability, and manual data reconciliation increases governance load, shifting the bottleneck from clinical decision-making to oversight and compliance. This represents an automation paradox where isolated digital acceleration degrades overall system performance.

The interconnected clinicians scenario (S_III_: low *A* and high *I*) describes an environment of high data interoperability but conservative AI automation. While data stewardship minimizes duplication and enables coordination, overall throughput remains capped by human cognitive limits and workforce availability.

Finally, the AI-orchestrated care system (S_IV_: high *A* and high *I*) integrates interoperable data with high AI adoption. This tends to remove upstream diagnostic constraints as they are more prone to algorithmic improvements. However, the system accelerates patient flow sufficiently to expose rigid downstream capacities in treatment delivery, and lower wait times induce further demand.

### Simulation With Emergent System Dynamics

To complement the qualitative scenario analysis, our DES captured higher-order dynamics and feedback loops such as governance work, virtual multidisciplinary team adoption, and induced demand (Figures S1A and S1B in Multimedia Appendix 3). The simulation also included operational strain due to increased throughput. As shown in Figure S1C in Multimedia Appendix 3, the high sensitivity of AI triage tools increased the false positive rate. This influx placed a disproportionate load on diagnostic resources, resulting in low-yield investigations that displaced confirmed cancer cases and competed for biopsy and histology capacity.

### Scenario Signatures and Performance Divergence

**Figure 2.**
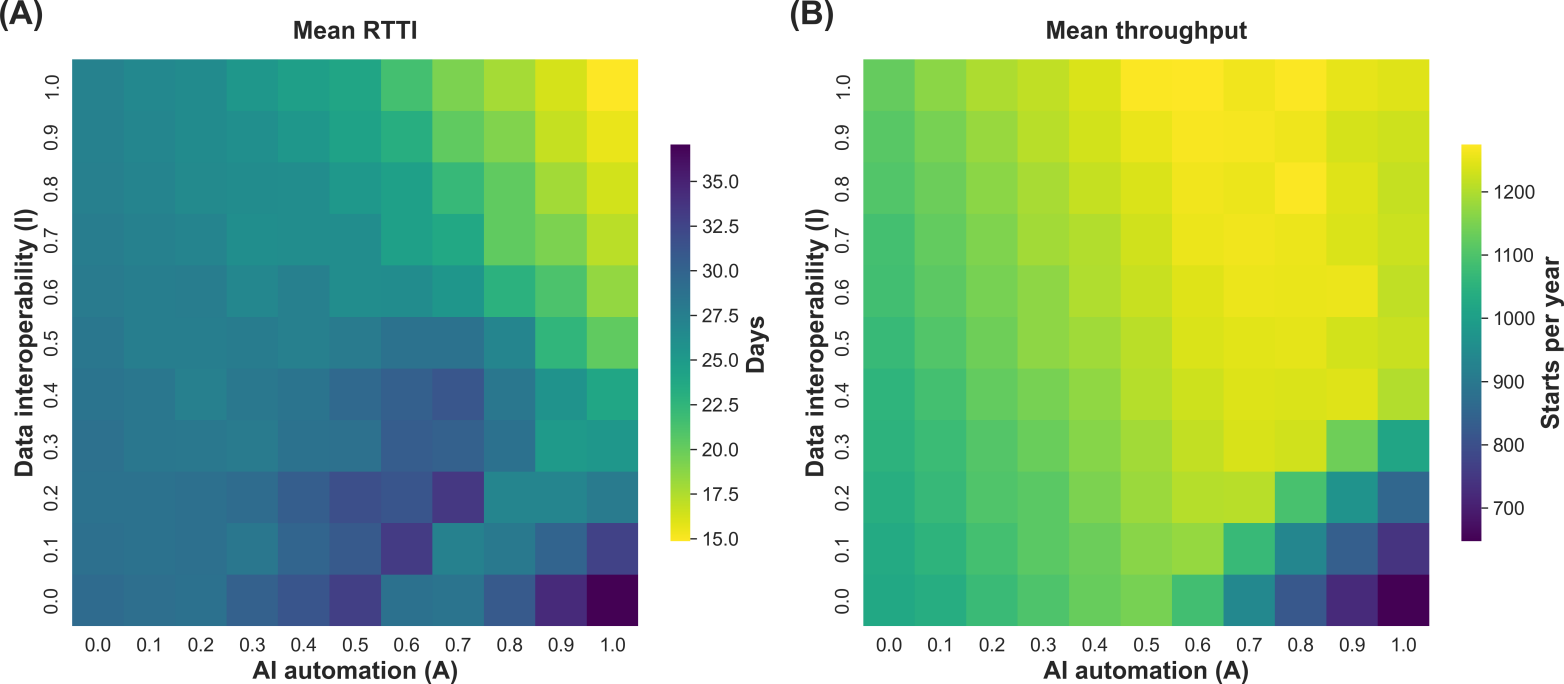
System-level performance indicators across the simulated adoption landscape. The heat maps illustrate the aggregate operational performance of the oncology pathways based on the discrete-event simulation. The x-axis represents artificial intelligence (AI) automation intensity, and the y-axis represents data interoperability, both normalized from 0.0 to 1.0. Panel (A) displays the mean referral-to-treatment interval measured in simulated days from patient intake to the initiation of definitive treatment. Panel (B) displays the annualized system throughput, quantified as the total number of treatment starts per year. All data points represent the average of 10 stochastic replications for each coordinate on the 11 × 11 parameter grid.

The simulation across the *A* × *I* parameter space generated distinct operational signatures for each scenario quadrant. As summarized in Table 1, the baseline (*S*_I_) functioned as a capacity-constrained system with a mean RTTI of 29.4 days and an annual throughput of 1022 patients. In the automation islands scenario (*S*_II_), we observed the worst system performance, with mean RTTI increasing to 37.1 days, a 26% increase compared to the analog baseline, whereas throughput decreased to 647 patients per year. Transitioning to interconnected clinicians (*S*_III_) yielded moderate improvements, reducing RTTI to 27.3 days. The AI-orchestrated care system (*S*_IV_) resulted in the highest patient flow, reducing the average RTTI to 14.9 days and increasing throughput to 1244 patients per year. Task-level improvements did not translate linearly into system performance (Figure 2). While the trajectory toward *S*_IV_ showed a combined increase in efficiency (lower RTTI) and number of treated patients, *S*_II_ resulted in higher RTTI and reduced throughput, suggesting that AI automation intensity may become counterproductive when isolated from data interoperability.

**Table 1.** Operational performance metric values by scenario representing the mean of 10 stochastic replications.

Scenario	*A* ^a^	*I* ^b^	RTTI^c^ (d), mean (SD)	Patients per year, mean (SD)	Primary bottleneck
S_I_: analog oncology	0.0	0.0	29.4 (0.5)	1022 (14.7)	CT^d^ scanner
S_II_: automation islands	1.0	0.0	37.1 (1.3)	647 (10.1)	Governance
S_III_: interconnected clinicians	0.0	1.0	27.3 (0.3)	1127 (14.0)	Molecular laboratory
S_IV_: AI^e^-orchestrated care	1.0	1.0	14.9 (0.3)	1244 (21.4)	Chemotherapy infusion units

a AI automation intensity variable.

b Data interoperability variable.

c RTTI: referral-to-treatment interval.

d CT: computed tomography.

e AI: artificial intelligence.

### Bottleneck Migration and Resource Dynamics

**Figure 3.**
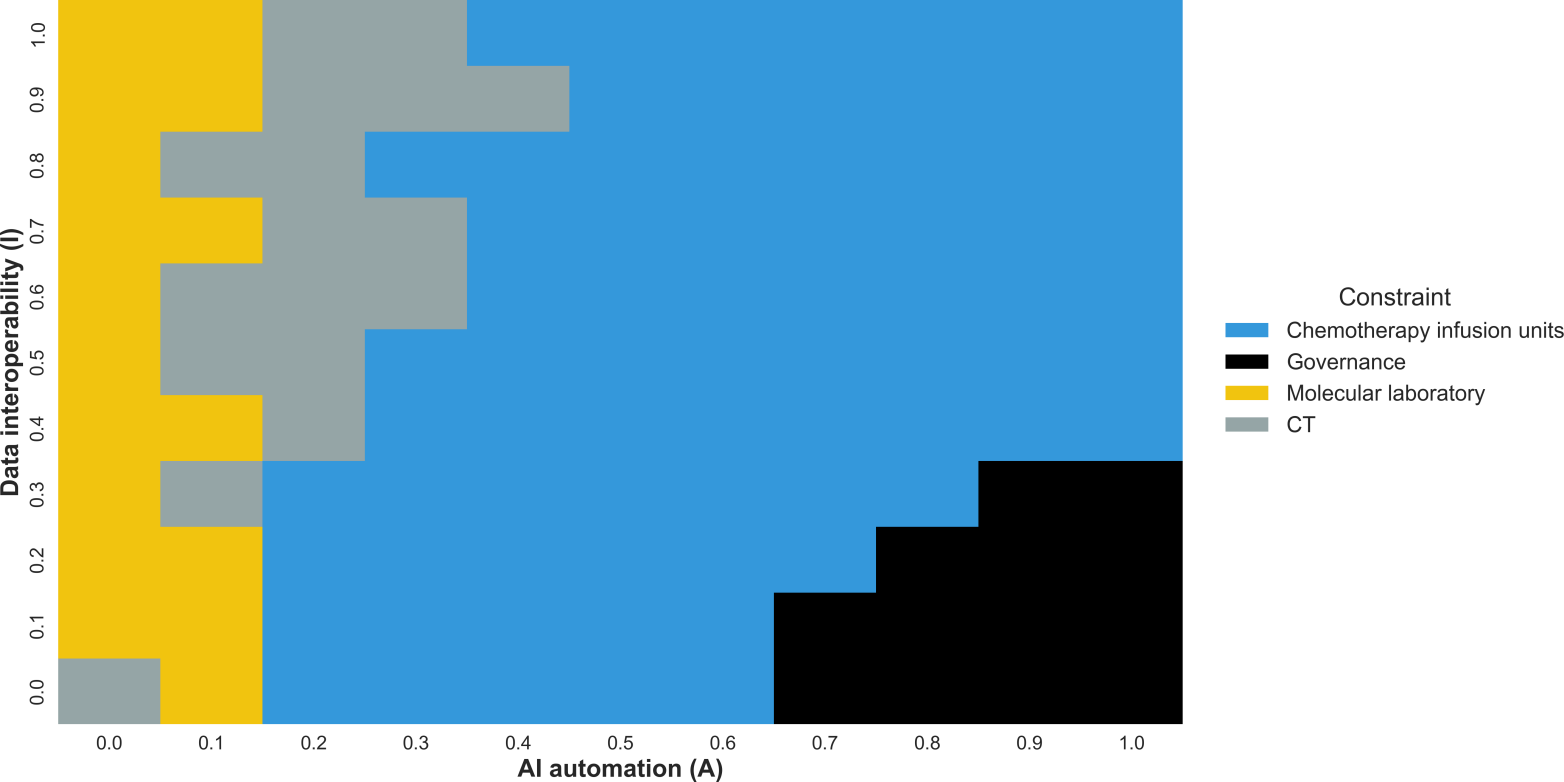
Structural bottleneck migration across the strategic state space: a categorical heat map of the primary binding constraint within the oncology pathway across the *A* × *I* scenario space. The x-axis represents artificial intelligence (AI) automation intensity, and the y-axis represents data interoperability, both normalized from 0.0 to 1.0. For each coordinate on the 11 × 11 grid, the simulation evaluates the operational load across all modeled clinical and administrative queues and identifies the specific resource pool with the highest mean use rate. CT: computed tomography.

Further analysis exposed a structural migration of constraints (Figure 3). In low–AI automation scenarios (S_I_ and S_III_), constraints were located upstream in diagnostics (computed tomography scanners and molecular laboratory). The automation islands (S_II_) scenario saw the primary constraint decouple from clinical infrastructure, shifting to administrative governance and quality assurance methods as manual oversight processes became overwhelmed. In the fully transformed S_IV_ scenario, the primary bottleneck shifted downstream to chemotherapy infusion units. Therefore, successful upstream AI adoption transferred capacity pressure from diagnostic infrastructure to labor-intensive treatment resources.

**Figure 4.**
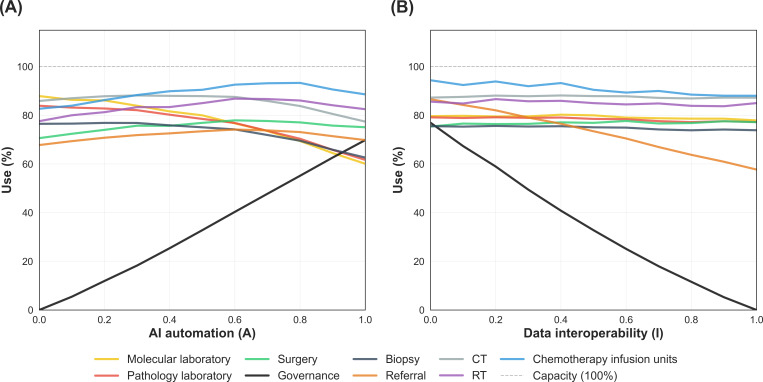
Resource use sensitivity analysis. The panels display the mean use rates of system resources derived from the discrete-event simulation. Panel (A) illustrates the impact of varying artificial intelligence (AI) automation intensity (*A*) from 0.0 to 1.0 while holding data interoperability (*I*) constant at a baseline of 0.5. Panel (B) demonstrates the effect of varying *I* with *A* fixed at 0.5. The dashed line represents the theoretical maximum capacity (100% use). CT: computed tomography; RT: radiotherapy.

To unpack these constraint shifts, we generated visualization tools that quantify how resource use changes across the *A* × *I* plane. Holding data interoperability constant while varying AI automation (Figure 4A) and holding AI automation constant while varying data interoperability (Figure 4B) yielded distinct use trajectories for each resource. Together, these plots identified crossover points at which the binding constraint moved from upstream diagnostics (computed tomography and laboratory services) to downstream treatment capacity (eg, chemotherapy, surgery, and radiotherapy) or administrative or digital functions (referral processing and governance), indicating where marginal investments likely had the greatest impact.

### Volatility and Optimal Pathways

**Figure 5.**
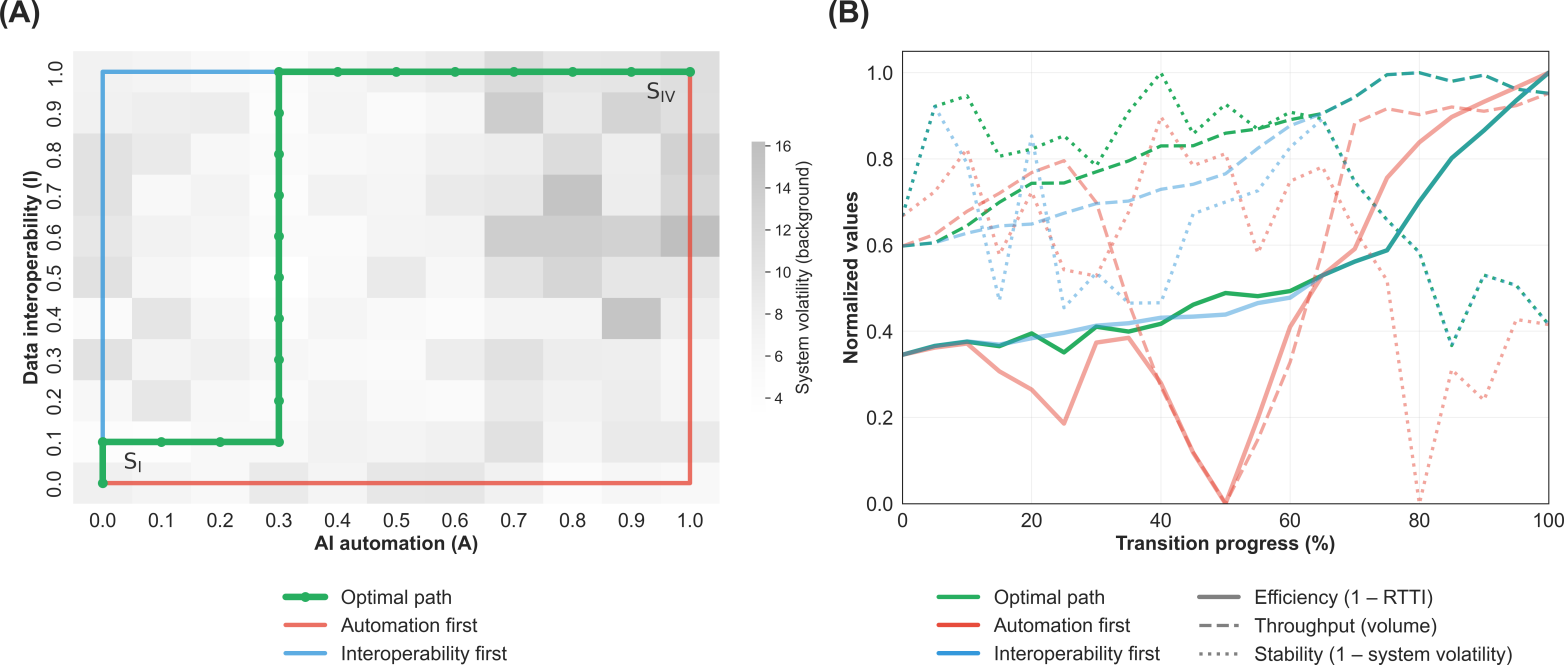
Pathway optimization and transition dynamics. Panel (A) presents a trajectory analysis across the artificial intelligence (AI) automation and data interoperability state space, modeled as a weighted directed graph. The background heat map indicates system volatility (defined as the mean of cross-replication SDs of the referral-to-treatment interval [RTTI] and annualized throughput), with darker regions representing high instability. The green line traces the computationally optimal path based on the weighted cost function, benchmarked against 2 alternative corner strategies: an automation-first approach (red line) and an interoperability-first approach (blue line). Panel (B) tracks key system metrics throughout the implementation process. Normalized metrics for efficiency (inverted RTTI), throughput volume, and stability (inverted system volatility) are plotted against transition progress (0%-100%).

Finally, we analyzed the SDs of the output parameters across 10 stochastic replications, treating volatility as a proxy for system stability. The SDs for RTTI and throughput were highest in the transition from S_II_ to S_IV_, indicating that uncoordinated AI adoption may create unpredictable wait times driven by sporadic governance backlogs (Figures S1D-F in Multimedia Appendix 3). In contrast, establishing high data interoperability first (S_III_) acted as a stabilizing force, allowing for the subsequent integration of AI automation. To determine the most robust trajectory from analog operations (S_I_) to the AI-orchestrated system (S_IV_), we performed a graph-based optimization minimizing a composite cost function of RTTI, throughput, and their respective operational variances (Figure 5A). The model selected an adoption sequence characterized by a structural preference for data interoperability–led scaling, prioritizing early gains in *I* ahead of *A*. This sequencing effectively reduced midtransition volatility, allowing the system to absorb the governance load of incremental AI automation while maintaining high throughput efficiency throughout the implementation life cycle (Figure 5B).

## Discussion

### Strategic Utility for Health System Planning

This paper addresses the gap between the rapid pace of technological transformation in medicine and the current underuse of applied futures methods, offering a novel approach to operational planning under uncertainty [20]. Traditional business cases for AI often rely on static efficiency assumptions, presuming that diagnostic time reductions linearly increase throughput. However, such linear extrapolations ignore endogenous feedback loops inherent in complex systems. By coupling scenario planning with quantified simulations, this study provides a framework to stress-test AI adoption strategies against plausible futures—making risks visible to health system planners before decisions are formed and capital is committed.

Because the model is parameterized around 2 fundamental strategic levers (AI automation intensity and data interoperability), it can inform both clinic-level operational redesign and broader health system–level investment and governance planning. To illustrate how health systems can operationalize this framework across different organizational levels, we have outlined 3 concrete implementation scenarios (Table S2 in Multimedia Appendix 2). These scenarios demonstrate how micro-level hospitals, meso-level regional networks, and macro-level state health authorities can use this framework to anticipate bottleneck migration, sequence investments, and mitigate transition volatility.

### Robust Strategies and Sequencing

A key insight derived from the scenario sweep is the phenomenon of bottleneck migration, necessitating a shift from local optimization to whole-system capacity planning. The simulation indicates that successful AI adoption in diagnostics tends to shift pressure downstream in the model. Therefore, executives must couple upstream AI investments with adaptive capacity planning for surgery, chemotherapy, and radiotherapy. Similarly, the emergence of higher false positive rates suggests that adoption strategies might require new guardrails such as confirmatory testing thresholds to prevent the system from being overwhelmed in scenarios of increased induced demand. At the same time, scenarios of high AI automation and low data interoperability should be avoided as they can lead to significant governance overload. This aligns with recent critiques of health workforce planning, which note that failure to account for social interactions and power dynamics, including governance, often undermines technical implementation [30].

Moreover, results from our pathway optimization demonstrated that a coordinated adoption strategy with early focus on data interoperability yielded the best overall results and stability. Because high volatility manifests in real-world settings as unpredictability and may lead to stakeholder resistance, implementation strategies must manage transition stability alongside performance optimization. This underlines the importance of sequencing—via phased rollouts or adaptive governance—to minimize disturbances during transformation. Conceptually, this optimization mirrored a backcasting approach, working backward from a desired end state to identify near-term moves that preserve system stability [31].

### Limitations and Future Directions

This study presents a mechanism-oriented framework prioritizing structural behavior over precise numerical forecasting. Importantly, the simulation is based on stylized parameters rather than real-world data. As an exploratory proof of concept, it has several limitations. First, parameters are based on conceptual assumptions rather than site-level empirical data, limiting the generalizability of specific point estimates. Alternative parameterizations could yield different quantitative results; thus, findings should be interpreted as directional hypotheses rather than definitive empirical conclusions. Second, the model is restricted to 3 tumor types and 2 stages, which does not capture the full complexity of a clinical case mix. Third, the simulation excludes financial costs, clinical end points, and equity impacts. Finally, adoption is modeled as a static state rather than a time-varying implementation process, simplifying the realities of organizational learning and change management.

To support applied decision-making, future work should calibrate parameters to local workflow distributions and resource profiles and validate model behavior against observed pathway metrics where available to assess external validity. Planned extensions include formal sensitivity and uncertainty analyses over capacities, service time assumptions, and objective function weights used in the transition optimization. Equity should be incorporated explicitly by stratifying patient agents by sociodemographic risk factors (eg, deprivation, language barriers, and digital access) and modeling differential access, adherence, and delay mechanisms to test whether accelerated pathways widen disparities [32,33]. Additional behavioral realism could be introduced by modeling clinician oversight burden, trust calibration, and AI literacy as determinants of effective automation and governance load [34,35].

### Conclusions

The integration of qualitative scenario planning with quantitative simulations offers a tool for testing plausible oncology AI strategies under uncertainty. AI can dramatically improve oncology operations but only when paired with interoperable data infrastructure and system-level capacity planning; otherwise, it risks worsening delays and instability. Simulating interactions between AI automation and data interoperability exposed operational tipping points, bottlenecks, and instabilities invisible to static planning models. Future research should extend this hybrid framework and integrate financial and equity dimensions to better anticipate the societal costs of technological disruption. This could help establish simulation-based scenario planning as an important tool in medical futures studies.

## Supplementary material

10.2196/92642 Multimedia Appendix 1 Code for discrete-event simulation.

10.2196/92642 Multimedia Appendix 2 Detailed operational mechanisms of the discrete-event simulation and applied case scenarios for operationalizing the framework.

10.2196/92642 Multimedia Appendix 3 Secondary operational metrics and uncertainty surfaces; visualization of the mechanism-based outputs for governance load (administrative audits per patient arrival), virtual multidisciplinary team adoption rates, and the false positive rate (defined as the proportion of biopsies triggered via artificial intelligence triage that results in a benign finding); and SDs of key performance indicators across 10 stochastic replications.

## Abbreviations

AI: artificial intelligence
DECIDE-AI: Developmental and Exploratory Clinical Investigations of Decision Support Systems Driven by Artificial Intelligence
DES: discrete-event simulation
RTTI: referral-to-treatment interval

## References

[1] May P, Greß J, Seidel C (2025). Enabling just-in-time clinical oncology analysis with large language models: feasibility and validation study using unstructured synthetic data. JMIR Med Inform.

[2] Chen D, Alnassar SA, Avison KE, Huang RS, Raman S (2025). Large language model applications for health information extraction in oncology: scoping review. JMIR Cancer.

[3] Lammert J, Pfarr N, Kuligin L (2025). Large language models-enabled digital twins for precision medicine in rare gynecological tumors. NPJ Digit Med.

[4] May P, Nokodian S, Nuernbergk C (2026). Artificial intelligence-assisted error detection in complex clinical documentation: leveraging large language models to enhance patient safety in oncology. JCO Clin Cancer Inform.

[5] Ferber D, El Nahhas OSM, Wölflein G (2025). Development and validation of an autonomous artificial intelligence agent for clinical decision-making in oncology. Nat Cancer.

[6] Chow R, Midroni J, Kaur J (2023). Use of artificial intelligence for cancer clinical trial enrollment: a systematic review and meta-analysis. J Natl Cancer Inst.

[7] Bandi A, Kongari B, Naguru R, Pasnoor S, Vilipala SV (2025). The rise of agentic AI: a review of definitions, frameworks, architectures, applications, evaluation metrics, and challenges. Future Internet.

[8] Karunanayake N (2025). Next-generation agentic AI for transforming healthcare. Inform Health.

[9] Carl N, Schramm F, Haggenmüller S (2024). Large language model use in clinical oncology. NPJ Precis Oncol.

[10] Ringeval M, Etindele Sosso FA, Cousineau M, Paré G (2025). Advancing health care with digital twins: meta-review of applications and implementation challenges. J Med Internet Res.

[11] Hao Y, Qiu Z, Holmes J (2025). Large language model integrations in cancer decision-making: a systematic review and meta-analysis. NPJ Digit Med.

[12] Huhulea EN, Huang L, Eng S (2025). Artificial intelligence advancements in oncology: a review of current trends and future directions. Biomedicines.

[13] Katonai G, Arvai N, Mesko B (2025). AI and primary care: scoping review. J Med Internet Res.

[14] Patel MS, Dee EC, Lee NY (2026). AI and human expertise in cancer care - striving for synergy. Nat Rev Clin Oncol.

[15] Stevens ER, Alfaro Arias V, Luu S, Lawrence K, Groom L (2025). Technology integration to support nurses in an “inpatient room of the future”: qualitative analysis. J Med Internet Res.

[16] McCann L, Lewis L, Oduntan O (2024). Patients’ and clinicians’ experiences using a real-time remote monitoring system for chemotherapy symptom management (ASyMS): qualitative study. J Med Internet Res.

[17] Meskó B, Drobni Z, Bényei É, Gergely B, Győrffy Z (2017). Digital health is a cultural transformation of traditional healthcare. Mhealth.

[18] Meskó B, Topol EJ (2023). The imperative for regulatory oversight of large language models (or generative AI) in healthcare. NPJ Digit Med.

[19] Vasey B, Nagendran M, Campbell B (2022). Reporting guideline for the early stage clinical evaluation of decision support systems driven by artificial intelligence: DECIDE-AI. BMJ.

[20] Meskó B, Kristóf T, Dhunnoo P, Árvai N, Katonai G (2024). Exploring the need for medical futures studies: insights from a scoping review of health care foresight. J Med Internet Res.

[21] Mesko B, Kristóf T, Dhunnoo P, Árvai N, Katonai G (2025). A practical guide to using futures methods in health care: approaches, applications, and case studies. J Med Internet Res.

[22] Mesko B (2024). The emergence of medical futures studies uncovers medicine and healthcare’s untapped potential. NPJ Digit Med.

[23] Schoemaker PJ (1995). Scenario planning: a tool for strategic thinking. Sloan Manag Rev.

[24] Gross M, Dewan A, Sabharwal K (2025). Decentralized biobanking pathway to precision medicine: futures study. J Med Internet Res.

[25] Giunti G, Glynn R, Hennessy J, Doherty CP (2025). Participatory design approach in the use of scenario analysis for futureproofing medical education: case study. J Med Internet Res.

[26] Babashov V, Aivas I, Begen MA (2017). Reducing patient waiting times for radiation therapy and improving the treatment planning process: a discrete-event simulation model (radiation treatment planning). Clin Oncol (R Coll Radiol).

[27] Dijkstra EW (1959). A note on two problems in connexion with graphs. Numer Math.

[28] Hagberg AA, Schult DA, Swart P, Varoquaux G, Vaught T, Millman J (2008). Proceedings of 7th Python in Science Conference (SciPy2008).

[29] (2023). Satzung der Ethikkommission der Technischen Universität München – nicht-medizinische Fachgruppe. Technische Universität München.

[30] Rees GH, Crampton P, Gauld R, MacDonell S (2018). Rethinking health workforce planning: capturing health system social and power interactions through actor analysis. Futures.

[31] Dreborg KH (1996). Essence of backcasting. Futures.

[32] Norris RP, Fuller E, Greystoke A, Todd A, Sharp L (2025). Routes to diagnosis in lung cancer-do socio-demographics matter? An English population-based study. Cancers (Basel).

[33] Gerrard AD, Coxon J, Maeda Y, Theodoratou E, Dunlop MG, Din FV (2024). Colorectal cancer prevalence in faecal immunochemical test non-returners: potential for health inequality in symptomatic referral pathways. BJS Open.

[34] Arvai N, Katonai G, Mesko B (2025). Health care professionals’ concerns about medical AI and psychological barriers and strategies for successful implementation: scoping review. J Med Internet Res.

[35] Meskó B (2023). Prompt engineering as an important emerging skill for medical professionals: tutorial. J Med Internet Res.

