# Supplementary material for: Sequencing AI Automation and Data Interoperability in Oncology Using a Scenario-Planning Framework Coupled With Discrete-Event Simulation: Proof-of-Concept Study
Source: J Med Internet Res. 2026 May 25;28:e92642. doi: 10.2196/92642 (PMC13200774; doi:10.2196/92642)
Supplement: Multimedia Appendix 1 [file jmir-v28-e92642-s001.docx]

import simpy

import random

import numpy as np

import pandas as pd

import matplotlib.pyplot as plt

import seaborn as sns

import matplotlib.colors as mcolors

import hashlib

from dataclasses import dataclass

from matplotlib.gridspec import GridSpec

from matplotlib.patches import Patch

class Calendar:

WORK_START = 8.0 / 24.0

WORK_END = 17.0 / 24.0

WORK_LEN = WORK_END - WORK_START

@staticmethod

def get_next_start_time(current_time: float) -> float:

d = int(current_time)

t = current_time % 1.0

day_idx = d % 7

if day_idx >= 5:

return float(d + (7 - day_idx)) + Calendar.WORK_START

if t >= Calendar.WORK_END:

days_to_add = 3 if day_idx == 4 else 1

return float(d + days_to_add) + Calendar.WORK_START

if t < Calendar.WORK_START:

return float(d) + Calendar.WORK_START

return current_time

@staticmethod

def get_completion_time(start_time: float, duration_days: float) -> float:

t = start_time

rem = duration_days

t = Calendar.get_next_start_time(t)

time_in_day = t % 1.0

can_do_today = Calendar.WORK_END - time_in_day

if rem <= can_do_today:

return t + rem

rem -= can_do_today

t = int(t) + 1.0

full_days = int(rem // Calendar.WORK_LEN)

rem_part = rem % Calendar.WORK_LEN

weeks = full_days // 5

days = full_days % 5

t += weeks * 7.0

while days > 0:

t = Calendar.get_next_start_time(t)

t += 1.0

days -= 1

t = Calendar.get_next_start_time(t)

t += rem_part

return t

@dataclass

class GlobalParams:

horizon_days: float = 365.0 * 3

warmup_days: float = 150.0

arrival_cutoff: float = horizon_days - 100.0

n_patients: int = 15000

base_interarrival: float = 0.45

prob_lung: float = 0.40

prob_breast: float = 0.40

prob_crc: float = 0.20

prob_late_stage: float = 0.40

class OncologySystem:

def __init__(self, env: simpy.Environment, A: float, I: float):

self.env = env

self.A = A

self.I = I

self.res = {

'referral_admin': simpy.Resource(env, capacity=2),

'governance_officer': simpy.Resource(env, capacity=1),

'scanner_ct': simpy.Resource(env, capacity=1),

'biopsy_suite': simpy.Resource(env, capacity=3),

'lab_histology': simpy.Resource(env, capacity=3),

'lab_molecular': simpy.Resource(env, capacity=3),

'oncologist': simpy.Resource(env, capacity=4),

'or_surgery': simpy.Resource(env, capacity=3),

'chemo_chair': simpy.Resource(env, capacity=4),

'linac_rt': simpy.Resource(env, capacity=3),

}

self.busy_times = {k: 0.0 for k in self.res}

self.total_arrivals = 0

self.total_biopsies = 0

self.patients_started_tx = 0

self.rework_events = 0

self.false_positive_events = 0

self.virtual_mdt_events = 0

self.governance_events = 0

self.patient_data = []

self.observed_dx_times = []

def get_service_time(self, base_days: float, ai_impact: float = 0.0, is_admin: bool = False) -> float:

ai_factor = 1.0 - (ai_impact * self.A)

i_impact = 0.4 if is_admin else 0.1

interop_factor = 1.0 - (i_impact * self.I)

return base_days * ai_factor * interop_factor

def request_task(self, res_name, base_duration, ai_impact=0.0, is_24_7=False, is_admin=False, atomic=False, record_dict=None, record_key=None):

active_duration = self.get_service_time(base_duration, ai_impact, is_admin)

if atomic and not is_24_7:

t_start = Calendar.get_next_start_time(self.env.now)

t_mod = t_start % 1.0

time_left_in_shift = Calendar.WORK_END - t_mod

if active_duration > time_left_in_shift:

t_start = Calendar.get_next_start_time(int(t_start) + 1.0)

if t_start > self.env.now:

yield self.env.timeout(t_start - self.env.now)

with self.res[res_name].request() as req:

yield req

if record_dict is not None and record_key is not None:

record_dict[record_key] = self.env.now

start_time = self.env.now

if is_24_7:

elapsed_duration = active_duration

else:

finish_time = Calendar.get_completion_time(start_time, active_duration)

elapsed_duration = finish_time - start_time

if self.env.now > GlobalParams.warmup_days:

end_of_task = self.env.now + active_duration

cutoff = min(end_of_task, GlobalParams.horizon_days)

start_count = max(self.env.now, GlobalParams.warmup_days)

if cutoff > start_count:

self.busy_times[res_name] += (cutoff - start_count)

yield self.env.timeout(elapsed_duration)

def patient_journey(env: simpy.Environment, pid: int, sys: OncologySystem):

arrival = env.now

if env.now > GlobalParams.warmup_days:

sys.total_arrivals += 1

patient_decision_delay = random.uniform(1, 4)

biopsy_recovery_delay = random.uniform(1, 3)

prehab_delay = random.uniform(2, 5)

stage_shift_factor = 0.05 * sys.A * sys.I

is_late = random.random() < (GlobalParams.prob_late_stage - stage_shift_factor)

stage = 'Late' if is_late else 'Early'

r = random.random()

if r < 0.4: cancer = 'Lung'

elif r < 0.8: cancer = 'Breast'

else: cancer = 'CRC'

yield from sys.request_task('referral_admin', 0.15, ai_impact=0.2, is_admin=True)

check_prob = sys.A * (1.0 - sys.I)

if random.random() < check_prob:

yield from sys.request_task('governance_officer', 0.12, ai_impact=0.0, is_admin=True)

if env.now > GlobalParams.warmup_days: sys.governance_events += 1

yield from sys.request_task('scanner_ct', 0.08, ai_impact=0.3, atomic=True)

fp_prob = 0.05 + (0.1 * sys.A)

if random.random() < fp_prob:

if env.now > GlobalParams.warmup_days:

sys.false_positive_events += 1

sys.total_biopsies += 1

yield from sys.request_task('biopsy_suite', 0.20, ai_impact=0.35, atomic=True)

yield from sys.request_task('lab_histology', 0.8, ai_impact=0.4, is_24_7=True)

return

if env.now > GlobalParams.warmup_days: sys.total_biopsies += 1

yield from sys.request_task('biopsy_suite', 0.20, ai_impact=0.35, atomic=True)

yield env.timeout(biopsy_recovery_delay)

while True:

yield from sys.request_task('lab_histology', 0.8, ai_impact=0.4, is_24_7=True)

base_fail = 0.1

fail_prob = base_fail * (1.0 - 0.5 * sys.A) * (1.0 - 0.3 * sys.I)

if random.random() < fail_prob:

if env.now > GlobalParams.warmup_days:

sys.rework_events += 1

sys.total_biopsies += 1

yield env.timeout(2.0)

yield from sys.request_task('biopsy_suite', 0.15, atomic=True)

else:

break

if (cancer == 'Lung') or (cancer == 'CRC' and stage == 'Late'):

yield from sys.request_task('lab_molecular', 2, ai_impact=0.4, is_24_7=True)

time_dx = env.now

if env.now > GlobalParams.warmup_days:

sys.observed_dx_times.append(time_dx - arrival)

prob_virtual = 0.9 * sys.A * sys.A * sys.I

if random.random() < prob_virtual:

if env.now > GlobalParams.warmup_days: sys.virtual_mdt_events += 1

yield from sys.request_task('oncologist', 0.05, ai_impact=0.1)

else:

day_of_week = int(env.now) % 7

days_to_mdt = (4 - day_of_week) % 7

if days_to_mdt == 0 and (env.now % 1.0) > 0.5: days_to_mdt = 7

yield env.timeout(days_to_mdt)

yield from sys.request_task('oncologist', 0.15, ai_impact=0.1)

yield env.timeout(patient_decision_delay + prehab_delay)

modality = []

if cancer == 'Breast':

modality = ['Surgery', 'RT'] if stage == 'Early' else ['Chemo', 'RT']

elif cancer == 'Lung':

modality = ['Surgery'] if stage == 'Early' else ['Chemo', 'RT']

elif cancer == 'CRC':

modality = ['Surgery'] if stage == 'Early' else ['Surgery', 'Chemo']

metrics = {}

for i, mod in enumerate(modality):

if i > 0: yield env.timeout(2.0)

rec_dict = metrics if i == 0 else None

rec_key = 'tx_start' if i == 0 else None

if mod == 'Surgery':

yield from sys.request_task('or_surgery', 0.3, ai_impact=0.1, atomic=True, record_dict=rec_dict, record_key=rec_key)

elif mod == 'Chemo':

yield from sys.request_task('chemo_chair', 0.8, ai_impact=0.0, atomic=True, record_dict=rec_dict, record_key=rec_key)

elif mod == 'RT':

yield from sys.request_task('linac_rt', 0.40, ai_impact=0.05, atomic=True, record_dict=rec_dict, record_key=rec_key)

if env.now > GlobalParams.warmup_days and 'tx_start' in metrics:

sys.patients_started_tx += 1

rtti = metrics['tx_start'] - arrival

sys.patient_data.append({

'RTTI': rtti,

'Time_to_Dx': time_dx - arrival

})

def run_scenario(A, I, seed_tuple):

seed_str = f"{seed_tuple[0]}-{seed_tuple[1]}-{seed_tuple[2]}"

secure_seed = int(hashlib.sha256(seed_str.encode()).hexdigest()[:8], 16)

random.seed(secure_seed)

env = simpy.Environment()

sys = OncologySystem(env, A, I)

def gen():

window_size = 50

while True:

if env.now > GlobalParams.arrival_cutoff:

break

current_ia = GlobalParams.base_interarrival

if len(sys.observed_dx_times) > window_size:

recent_avg = np.mean(sys.observed_dx_times[-window_size:])

target_dx = 21.0

ratio = recent_avg / target_dx

demand_factor = max(0.5, min(1.5, ratio ** 1.1))

current_ia = current_ia * demand_factor

yield env.timeout(random.expovariate(1.0 / current_ia))

env.process(patient_journey(env, 0, sys))

env.process(gen())

env.run(until=GlobalParams.horizon_days)

obs_time = GlobalParams.horizon_days - GlobalParams.warmup_days

loads = {}

for name, busy_t in sys.busy_times.items():

cap = sys.res[name].capacity

is_24_7 = 'lab' in name

if is_24_7:

avail_time = obs_time * cap

else:

work_factor = (5 * 9) / (7 * 24)

avail_time = obs_time * cap * work_factor

loads[f"load_{name}"] = min(1.0, busy_t / avail_time) if avail_time > 0 else 0

df = pd.DataFrame(sys.patient_data)

mean_rtti = df['RTTI'].mean() if not df.empty else 0

throughput = (sys.patients_started_tx / obs_time) * 365.0

burnout_risk = (sys.governance_events / sys.total_arrivals) if sys.total_arrivals > 0 else 0

denom_biopsy = sys.total_biopsies if sys.total_biopsies > 0 else 1

rework_rate = sys.rework_events / denom_biopsy

fp_rate = sys.false_positive_events / denom_biopsy

denom_tx = sys.patients_started_tx if sys.patients_started_tx > 0 else 1

virtual_rate = sys.virtual_mdt_events / denom_tx

max_load_val = max(loads.values())

bottleneck_name = [k for k, v in loads.items() if v == max_load_val][0].replace('load_', '')

return {

'RTTI': mean_rtti,

'Throughput_Starts': throughput,

'Rework_Rate': rework_rate,

'False_Positive_Rate': fp_rate,

'Burnout_Risk': burnout_risk,

'Virtual_MDT_Rate': virtual_rate,

'Bottleneck_Raw': bottleneck_name,

**loads

}

def run_sweep(resolution=11):

print(f"Running {resolution}x{resolution} simulation (Atomic Fix + Uncertainty)...")

results = []

vals = np.linspace(0, 1, resolution)

reps_count = 10

total_runs = len(vals) * len(vals) * reps_count

count = 0

for A in vals:

for I in vals:

batch_results = []

for r in range(reps_count):

batch_results.append(run_scenario(A, I, seed_tuple=(A, I, r)))

count += 1

df_batch = pd.DataFrame(batch_results)

avg_res = df_batch.mean(numeric_only=True).to_dict()

std_res = df_batch.std(numeric_only=True).add_suffix('_std').to_dict()

b_list = [br['Bottleneck_Raw'] for br in batch_results]

avg_res['Bottleneck'] = max(set(b_list), key=b_list.count)

final_res = {**avg_res, **std_res}

final_res['A'] = A

final_res['I'] = I

results.append(final_res)

if count % 50 == 0:

print(f"Progress: {count}/{total_runs}")

return pd.DataFrame(results)

def plot_and_save_dashboard(df):

df['A'] = df['A'].round(2)

df['I'] = df['I'].round(2)

print("Saving raw data to 'oncology_simulation_results.csv'...")

df.to_csv("oncology_simulation_results.csv", index=False)

sns.set_theme(style="white", context="paper")

FS_TITLE_MAIN = 28

FS_TITLE_SUB = 22

FS_LABEL = 18

FS_TICK = 16

FS_LEGEND = 18

def clean_heatmap(ax, val, title, cmap, cbar_label=None):

piv = df.pivot(index='I', columns='A', values=val).sort_index(ascending=False)

sns.heatmap(piv, ax=ax, cmap=cmap, cbar=True, annot=False,

cbar_kws={'shrink': 0.8, 'label': cbar_label if cbar_label else ''})

ax.set_title(title, fontweight='bold', fontsize=FS_TITLE_SUB, pad=15)

ax.set_xlabel("AI Automation (A)", fontsize=FS_LABEL)

ax.set_ylabel("Data Interoperability (I)", fontsize=FS_LABEL)

ax.tick_params(axis='x', which='major', labelsize=FS_TICK)

ax.tick_params(axis='y', which='major', labelsize=FS_TICK, rotation=0)

cbar = ax.collections[0].colorbar

cbar.ax.tick_params(labelsize=FS_TICK)

if cbar_label:

cbar.set_label(cbar_label, size=FS_LABEL)

print("Generating Image 1: Mean RTTI and Throughput...")

fig1 = plt.figure(figsize=(22, 10))

gs1 = GridSpec(1, 2, figure=fig1, wspace=0.3)

ax1_1 = fig1.add_subplot(gs1[0, 0])

clean_heatmap(ax1_1, 'RTTI', 'Mean RTTI', 'viridis_r', 'Days')

ax1_2 = fig1.add_subplot(gs1[0, 1])

clean_heatmap(ax1_2, 'Throughput_Starts', 'Mean Throughput', 'viridis', 'Starts/Year')

plt.savefig("1_KPIs.png", dpi=300, bbox_inches='tight')

plt.close(fig1)

print("Generating Image 2: Governance & Uncertainty...")

fig2 = plt.figure(figsize=(24, 16))

gs2 = GridSpec(2, 6, figure=fig2, hspace=0.4, wspace=0.4)

ax2_1 = fig2.add_subplot(gs2[0, 0:3])

clean_heatmap(ax2_1, 'Burnout_Risk', 'Governance Load', 'magma', 'Events/Arrival')

ax2_2 = fig2.add_subplot(gs2[0, 3:6])

clean_heatmap(ax2_2, 'Virtual_MDT_Rate', 'Virtual MDT Adoption', 'mako', 'Rate (0-1)')

ax2_3 = fig2.add_subplot(gs2[1, 0:2])

clean_heatmap(ax2_3, 'RTTI_std', 'Uncertainty: RTTI', 'cividis', 'Std Dev (Days)')

ax2_4 = fig2.add_subplot(gs2[1, 2:4])

clean_heatmap(ax2_4, 'Throughput_Starts_std', 'Uncertainty: Throughput', 'cividis', 'Std Dev (Starts/Yr)')

ax2_5 = fig2.add_subplot(gs2[1, 4:6])

clean_heatmap(ax2_5, 'Burnout_Risk_std', 'Uncertainty: Governance', 'cividis', 'Std Dev (Events)')

plt.savefig("2_Governance_Uncertainty.png", dpi=300, bbox_inches='tight')

plt.close(fig2)

print("Generating Image 3: Bottleneck Shift...")

fig3 = plt.figure(figsize=(14, 12))

ax3 = fig3.add_subplot(111)

res_meta = {

'lab_molecular': {'label': 'Molecular Lab', 'color': '#f1c40f'},

'lab_histology': {'label': 'Pathology Lab', 'color': '#e74c3c'},

'or_surgery': {'label': 'Surgery', 'color': '#2ecc71'},

'governance_officer': {'label': 'Governance', 'color': '#000000'},

'chemo_chair': {'label': 'Chemo', 'color': '#3498db'},

'biopsy_suite': {'label': 'Biopsy', 'color': '#34495e'},

'referral_admin': {'label': 'Referral', 'color': '#e67e22'},

'scanner_ct': {'label': 'CT', 'color': '#95a5a6'},

'linac_rt': {'label': 'RT', 'color': '#9b59b6'}

}

b_unique = sorted(df['Bottleneck'].unique())

b_map_int = {name: i for i, name in enumerate(b_unique)}

df['B_Int'] = df['Bottleneck'].map(b_map_int)

present_colors = []

for b in b_unique:

if b in res_meta:

present_colors.append(res_meta[b]['color'])

else:

present_colors.append('#000000')

cmap_disc = mcolors.ListedColormap(present_colors)

p_bn = df.pivot(index='I', columns='A', values='B_Int').sort_index(ascending=False)

sns.heatmap(p_bn, ax=ax3, cmap=cmap_disc, cbar=False)

ax3.set_title('Primary Bottleneck Shift', fontweight='bold', fontsize=FS_TITLE_SUB, pad=15)

ax3.set_xlabel("AI Automation (A)", fontsize=FS_LABEL)

ax3.set_ylabel("Data Interoperability (I)", fontsize=FS_LABEL)

ax3.tick_params(axis='x', labelsize=FS_TICK)

ax3.tick_params(axis='y', labelsize=FS_TICK, rotation=0)

legend_elements = []

for b in b_unique:

if b in res_meta:

lbl = res_meta[b]['label']

clr = res_meta[b]['color']

legend_elements.append(Patch(facecolor=clr, label=lbl))

ax3.legend(handles=legend_elements, title="Constraint", loc='center left',

bbox_to_anchor=(1.02, 0.5), fontsize=FS_LEGEND, title_fontsize=FS_LABEL)

plt.savefig("3_Bottleneck_Shift.png", dpi=300, bbox_inches='tight')

plt.close(fig3)

print("Generating Image 4: Resource Loads...")

fig4 = plt.figure(figsize=(24, 12))

gs4 = GridSpec(1, 2, figure=fig4, wspace=0.3, bottom=0.20)

ax4_1 = fig4.add_subplot(gs4[0, 0])

ax4_2 = fig4.add_subplot(gs4[0, 1])

resources_to_plot = [

('load_lab_molecular', 'Molecular Lab', '#f1c40f'),

('load_lab_histology', 'Pathology Lab', '#e74c3c'),

('load_or_surgery', 'Surgery', '#2ecc71'),

('load_governance_officer', 'Governance', '#000000'),

('load_chemo_chair','Chemo','#3498db'),

('load_biopsy_suite', 'Biopsy', '#34495e'),

('load_referral_admin', 'Referral', '#e67e22'),

('load_scanner_ct', 'CT', '#95a5a6'),

('load_linac_rt', 'RT', '#9b59b6')

]

mid_I = df.iloc[(df['I']-0.5).abs().argsort()[:1]]['I'].values[0]

slice_A = df[df['I'] == mid_I].sort_values('A')

for col_name, label, color in resources_to_plot:

ax4_1.plot(slice_A['A'], slice_A[col_name], label=label, color=color, linewidth=4, alpha=0.9)

ax4_1.axhline(y=1.0, color='gray', linestyle='--', alpha=0.5)

ax4_1.set_title(f'Resource Loads at Fixed Data Interoperability (I={mid_I})', fontweight='bold', fontsize=FS_TITLE_SUB)

ax4_1.set_xlabel('AI Automation (A)', fontsize=FS_LABEL)

ax4_1.set_ylabel('Utilization %', fontsize=FS_LABEL)

ax4_1.set_xlim(0, 1)

ax4_1.set_ylim(0, 1.1)

ax4_1.grid(True, alpha=0.3)

ax4_1.tick_params(axis='both', labelsize=FS_TICK, rotation=0)

mid_A = df.iloc[(df['A']-0.5).abs().argsort()[:1]]['A'].values[0]

slice_I = df[df['A'] == mid_A].sort_values('I')

for col_name, label, color in resources_to_plot:

ax4_2.plot(slice_I['I'], slice_I[col_name], label=label, color=color, linewidth=4, alpha=0.9)

ax4_2.axhline(y=1.0, color='gray', linestyle='--', alpha=0.5)

ax4_2.set_title(f'Resource Loads at Fixed AI Automation (A={mid_A})', fontweight='bold', fontsize=FS_TITLE_SUB)

ax4_2.set_xlabel('Data Interoperability (I)', fontsize=FS_LABEL)

ax4_2.set_ylabel('Utilization %', fontsize=FS_LABEL)

ax4_2.set_xlim(0, 1)

ax4_2.set_ylim(0, 1.1)

ax4_2.grid(True, alpha=0.3)

ax4_2.tick_params(axis='both', labelsize=FS_TICK, rotation=0)

handles, labels = ax4_1.get_legend_handles_labels()

fig4.legend(handles, labels, loc='lower center', bbox_to_anchor=(0.5, 0.02),

fancybox=True, shadow=False, ncol=5, fontsize=FS_LEGEND)

plt.savefig("4_Resource_Loads.png", dpi=300, bbox_inches='tight')

plt.close(fig4)

print("All 4 plots generated successfully.")

if __name__ == "__main__":

df_res = run_sweep(resolution=11)

plot_and_save_dashboard(df_res)
