# Supplementary material for: Sequencing AI Automation and Data Interoperability in Oncology Using a Scenario-Planning Framework Coupled With Discrete-Event Simulation: Proof-of-Concept Study
Source: J Med Internet Res. 2026 May 25;28:e92642. doi: 10.2196/92642 (PMC13200774; doi:10.2196/92642)
Supplement: Multimedia Appendix 3 [file jmir-v28-e92642-s003.docx]

**
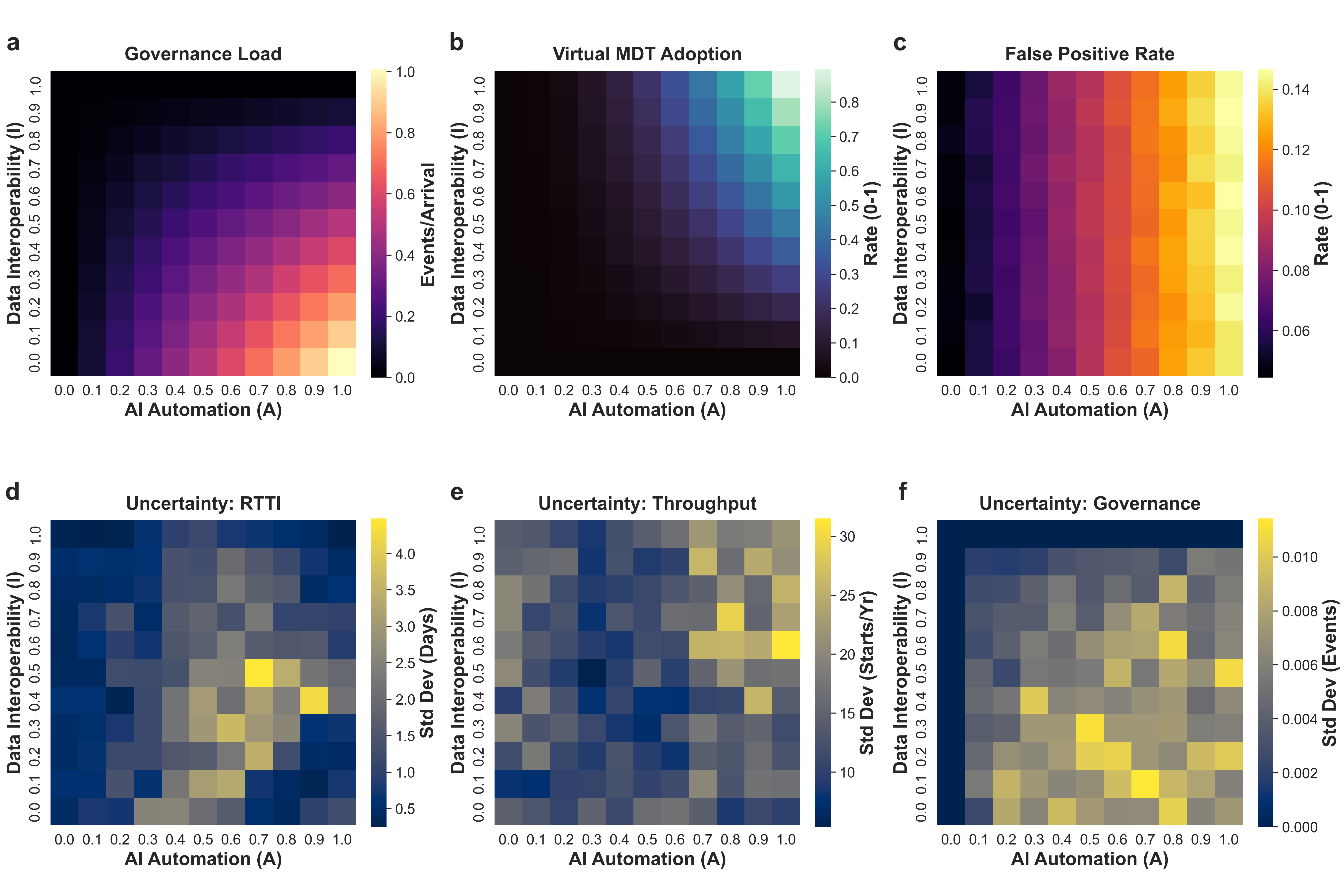
**

**Supplementary Figure S1. Secondary operational metrics and uncertainty surfaces.** **a-c)** Visualization of the mechanism-based outputs for governance load (administrative audits per patient arrival), virtual multidisciplinary team (MDT) adoption rates and the false-positive rate (defined as proportion of biopsies triggered by AI triage that results in a benign finding). **d-f)** Standard deviations of key performance indicators across n=10 stochastic replications.
